# Sequential hybrid ablation vs. surgical CryoMaze alone for treatment of atrial fibrillation: results of multicentre randomized controlled trial

**DOI:** 10.1093/europace/euae040

**Published:** 2024-02-02

**Authors:** Alan Bulava, Dan Wichterle, Aleš Mokráček, Pavel Osmančík, Petr Budera, Petr Kačer, Linda Vetešková, Petr Němec, Tomáš Skála, Petr Šantavý, Jan Chovančík, Piotr Branny, Vitalii Rizov, Miroslav Kolesár, Iva Šafaříková, Marian Rybář, for the SURHYB Trial Investigators, Alan Bulava, Aleš Mokráček, Jiří Haniš, Přemysl Hájek, Iva Šafaříková, David Sitek, Adam Novotný, Pavel Osmančík, Petr Kačer, Dalibor Heřman, Vitalii Rizov, Ondřej Süssenbek, Dan Wichterle, Petr Budera, Ondrej Szárszoi, Lukáš Salavec, Petr Peichl, Josef Kautzner, Ivan Netuka, Jiří Ondrášek, Linda Vetešková, Petr Němec, Jana Frantová, Tomáš Skála, Petr Šantavý, Dalibor Klimeš, Mariwan Majid, Miroslav Kolesár, Pavel Červinka, Jan Chovančík, Piotr Branny, Otakar Jiravský

**Affiliations:** Faculty of Health and Social Sciences, University of South Bohemia in České Budějovice and Cardiac Centre, České Budějovice Hospital, B. Němcové 54, 370 01 České Budějovice, Czechia; Cardiology Department, Institute for Clinical and Experimental Medicine, Prague, Czechia; Faculty of Health and Social Sciences, University of South Bohemia in České Budějovice and Cardiac Centre, České Budějovice Hospital, B. Němcové 54, 370 01 České Budějovice, Czechia; Third Faculty of Medicine, Charles University and University Hospital Královské Vinohrady, Prague, Czechia; Cardiology Department, Institute for Clinical and Experimental Medicine, Prague, Czechia; Third Faculty of Medicine, Charles University and University Hospital Královské Vinohrady, Prague, Czechia; Centre of Cardiovascular Surgery and Transplantation, Brno, Czechia; Centre of Cardiovascular Surgery and Transplantation, Brno, Czechia; Faculty of Medicine and Dentistry, Palacký University and University Hospital Olomouc, Olomouc, Czechia; Faculty of Medicine and Dentistry, Palacký University and University Hospital Olomouc, Olomouc, Czechia; Cardilogy Department, Hospital Agel Třinec—Podlesí, Třinec, Czechia; Cardilogy Department, Hospital Agel Třinec—Podlesí, Třinec, Czechia; Cardilogy Department, Masaryk Hospital, Ústí nad Labem, Czechia; Cardilogy Department, Masaryk Hospital, Ústí nad Labem, Czechia; Faculty of Health and Social Sciences, University of South Bohemia in České Budějovice and Cardiac Centre, České Budějovice Hospital, B. Němcové 54, 370 01 České Budějovice, Czechia; Faculty of Biomedical Engineering, Czech Technical University in Prague, Kladno, Czechia

**Keywords:** Atrial fibrillation, Catheter ablation, Concomitant atrial fibrillation ablation, Hybrid ablation, Maze procedure

## Abstract

**Aims:**

Data on the hybrid atrial fibrillation (AF) treatment are lacking in patients with structural heart disease undergoing concomitant CryoMaze procedures. The aim was to assess whether the timely pre-emptive catheter ablation would achieve higher freedom from AF or atrial tachycardia (AT) and be associated with better clinical outcomes than surgical ablation alone.

**Methods and results:**

The trial investigated patients with non-paroxysmal AF undergoing coronary artery bypass grafting and/or valve repair/replacement with mandatory concomitant CryoMaze procedure who were randomly assigned to undergo either radiofrequency catheter ablation [Hybrid Group (HG)] or no further treatment (Surgery Group). The primary efficacy endpoint was the first recurrence of AF/AT without class I or III antiarrhythmic drugs as assessed by implantable cardiac monitors. The primary clinical endpoint was a composite of hospitalization for arrhythmia recurrence, worsening of heart failure, cardioembolic event, or major bleeding. We analysed 113 and 116 patients in the Hybrid and Surgery Groups, respectively, with a median follow-up of 715 (IQR: 528–1072) days. The primary efficacy endpoint was significantly reduced in the HG [41.1% vs. 67.4%, hazard ratio (HR) = 0.38, 95% confidence interval (CI): 0.26–0.57, *P* < 0.001] as well as the primary clinical endpoint (19.9% vs. 40.1%, HR = 0.51, 95% CI: 0.29–0.86, *P* = 0.012). The trial groups did not differ in all-cause mortality (10.6% vs. 8.6%, HR = 1.17, 95%CI: 0.51–2.71, *P* = 0.71). The major complications of catheter ablation were infrequent (1.9%).

**Conclusion:**

Pre-emptively performed catheter ablation after the CryoMaze procedure was safe and associated with higher freedom from AF/AT and improved clinical outcomes.

## Graphical Abstract

**Graphical Abstract:**
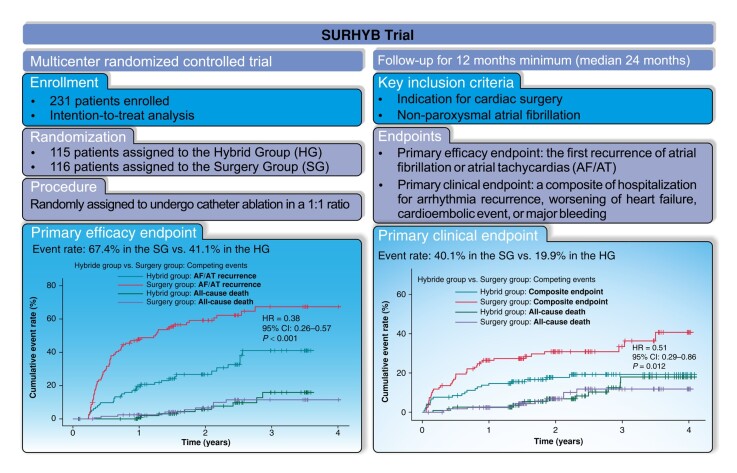

**What’s new?**

- Hybrid ablation strategy in patients undergoing concomitant CryoMaze procedure is associated with a 62% relative reduction of atrial fibrillation (AF)/atrial tachycardia (AT) recurrence and also with overall reduction of arrhythmia burden and occurrence of a persistent type of AF compared to CryoMaze alone.
- Pre-emptive catheter ablation in patients after concomitant CryoMaze procedure is associated with a 49% relative reduction of hospitalization for arrhythmia recurrence, worsening of heart failure, cardioembolic event, or significant bleeding.
- Pre-emptive catheter ablation in patients after concomitant CryoMaze procedure also reduces the risk for emergency out-patient visits for fibrillation/AT recurrence, for worsening of heart failure, and the need for either pharmacological or electrical cardioversions.

## Introduction

Atrial fibrillation (AF) is associated with increased mortality and morbidity.^1,2^ In patients indicated for cardiac surgery, the prevalence of AF is higher than in the general population and is as high as 50% in individuals with mitral valve disease.^3,4^ Moreover, AF is not only frequent in patients undergoing cardiac surgery but AF ablation is also of clinical benefit in patients with coronary artery disease (CAD). Indeed, a recent study demonstrated that AF ablation in ischaemic patients should not be denied, as CAD and revascularisation were not independent predictors of recurrence after ablation.^5^

Both surgical and catheter ablations are nowadays recognized AF treatments. Surgical ablation consists of a predefined set of lesions in both atria (the Cox-Maze IV procedure). Currently, the procedure is performed using either radiofrequency (RF) energy (bipolar RF clamps or unipolar RF pen) or cryothermal tissue destruction (CryoMaze).^6^ Typically, the CryoMaze is done in conjunction (i.e. as a concomitant procedure) with a coronary artery bypass or valve surgery.

The efficacy of the concomitant CryoMaze procedure as a treatment for persistent AF has been demonstrated in several studies.^7^ Still, the results were somewhat marginalized by small numbers of patients and far from optimum follow-up assessment of heart rhythm, often only by telephone interviews with occasional Holter recordings.^4,8^ Inconsistent extent and quality of rhythm monitoring have led to discordant ‘freedom from AF’ rates reported between 47 and 95%.^9,10^ Moreover, incomplete lines after CryoMaze are not infrequent, and conduction gaps could be pro-arrhythmic.^11,12^ The lines can be mapped and completed during subsequent transvenous RF catheter ablation (RFCA), however, the clinical effect of RFCA performed on top of previous surgical procedures is unknown. Thus, no recommendations regarding the most effective treatment strategy for post-CryoMaze patients are available.

In our previous non-randomized study in patients with non-paroxysmal AF, concomitant surgical CryoMaze procedures followed by RFCA (i.e. sequential hybrid strategy) led to excellent rhythm outcomes.^13^ The reason was the high rate of conduction gaps after the surgical procedure: complete pulmonary vein isolation (PVI) and posterior left atrial wall isolation were present in only 66 and 51% of all patients, respectively, and were completed in all patients by touch-up RFCA. Therefore, staged RFCA, which involves closing the conduction gaps in the surgical lesion set and eliminating any remaining arrhythmogenic substrate, would address the limitations of the isolated CryoMaze procedure. Such an approach would be associated with superior rhythm control and more favourable patient clinical outcomes. This hypothesis was investigated in Sequential **HYB**rid Ablation vs. **SUR**gical CryoMaze Alone for Treatment of Atrial Fibrillation Trial (SURHYB Trial).

## Methods

### Trial design

The SURHYB Trial was conducted as an investigator-initiated, multicentre, open-label, parallel-group, randomized controlled trial in seven major complex cardiovascular centres in Czechia. The details of the trial design have been published.^14^ The trial protocol was approved by the institutional ethics committees at all participating institutions. The trial followed the Helsinki Declaration of 1964, its later amendments, and the Good Clinical Practice Guidelines. Written informed consent was obtained from all patients before enrolment. The trial was designed and overseen by a Trial Steering Committee. An independent Data and Safety Monitoring Board guided the trial. All relevant events were adjudicated by an independent Endpoint Review Committee. Details of the participating centres and committee members are provided in the Supplementary material online, *Appendix*.

The SURHYB Trial was sponsored by the Czech Ministry of Health via a research grant from the Czech Health Research Council (registration No NV19-02-00046). The regulations regarding medical confidentiality and data protection were fulfilled. The trial was registered in the Czech Clinical Trials Registry, cz-020420181253 (accessible at www.ablace.cz).

### Trial population

Patients older than 18 years with symptomatic, non-paroxysmal AF documented on 12-lead-ECG who were indicated for cardiac surgery (coronary artery bypass grafting, valve surgery, or a combination of both) were screened. They were eligible for the trial if suitable for the concomitant CryoMaze procedure based on expert consensus statement.^15^ Exclusion criteria were AF secondary to a reversible cause, left atrial diameter (in parasternal long axis view) >55 mm, previous surgical or catheter ablation for AF or atrial tachycardia (AT), chronic kidney disease (Stage ≥4), contraindication to systemic anticoagulation, estimated life expectancy <1 year, and inability to mentally/physically comply with all trial requirements. Patients were screened for the trial before the surgery, but the randomization was done after the surgical procedure.

### Trial intervention and randomization

The CryoMaze procedure was carried out using nitrous oxide (N_2_0)-based cryoablation with the aluminium cryoICE ablation probe (AtriCure, Inc., Cincinnati, Ohio, United States) or the argon-based cryoablation with the stainless steel Cardioblate CryoFlexTM 10-S probe (Medtronic, Inc., Minneapolis, Minnesota, United States). The protocol consisted of mandatory circular lesions around the ipsilateral right and left pulmonary veins with linear lesions between the superior and inferior pulmonary veins to isolate the left atrium (LA) posterior wall. A mitral isthmus ablation line was created in all patients from the inferior connecting lesion towards the mitral annulus. Epicardial ablation of the coronary sinus contralateral to the mitral isthmus line was mandated. In addition, the ligament of Marshall was cut off in all patients, and a connecting line between the left superior pulmonary vein and the base of the left atrial appendage was created (see Supplementary material online, *Figure S1*). The LA appendage was excluded in patients with a CHA_2_DS_2_-VASc score ≥2. The right atrium cryolesions were performed at the discretion of the surgeon. Such lesions may have included but were not limited to superior/inferior vena cava isolation, intercaval lesion, and cavotricuspid isthmus (CTI) lesion. An implantable cardiac monitor (ICM) with telemonitoring capabilities and everyday ECG transmission (Biomonitor 2-AF and later Biomonitor III, Biotronik, Germany) was implanted before hospital discharge. The telemonitoring function of the device was enabled, and the patient unit (Cardiomessenger) was distributed to all patients.

Patients were randomly assigned in a 1:1 ratio to (i) **the Hybrid Group (HG)** or (ii) **the Surgery Group (SG)**. Randomization was performed post-operatively within 7 days after surgery but before hospital discharge, whichever occurred earlier. This ensured that the surgeons performing CryoMaze were unaware of treatment group allocation at the time of surgery. Covariate-adaptive randomization was implemented within the web-based electronic case report form. It was used to ensure the balance between treatment groups in the type of surgery (open/closed-heart) and the CHA_2_DS_2_-VASc score in three categories: (i) 0–2, (ii) 3–5, and (iii) 6–9.

Patients randomized to the HG were admitted for a staged RFCA 90 (70–110) days after the surgical procedure. Dense electroanatomic mapping of the left and right atria was performed using a CARTO3 navigation system and a Thermocool SmartTouch^®^ ablation catheter (Biosense Webster, Inc., USA) to provide information about the location of the cryolesions. The multipolar circular mapping catheter (Lasso™, Biosense Webster, Inc., USA) was used in all pulmonary vein antra to detect the localization of electrical reconnections. Signal recordings in sinus rhythm and during standard pacing maneuvers were used to identify the conduction gaps correctly. The same catheter was also placed on the posterior left atrial wall and at the base of the appendage remnant for pacing maneuvers to detect mitral isthmus conduction block and the LA posterior wall isolation, respectively. The goal was to close all gaps in circular and linear lines using RF energy and create a CTI block. A maximum energy of 30W with an ablation index not exceeding 450 was allowed on the posterior wall. In contrast, in all other locations, the RF energy could be up-titrated to 35 W with a maximum ablation index of 600. A recommended contact force should be above 10 g but not exceeding 30 g. Finally, all procedural atrial tachycardias (ATs), spontaneous or induced, were mapped and ablated. We did not use an oesophageal temperature probe in any patient.

Participants in the SG (control group) received surgical CryoMaze only. RFCA in the HG and CryoMaze in the SG were considered **index procedures** for primary efficacy endpoint analysis.

### Follow-up

During a 3-month blanking period **following the index procedure**, any treatment was allowed to maintain normal sinus rhythm (i.e. electrical or pharmacological cardioversion, antiarrhythmic drugs (AADs) initiation or dose escalation), except repeated RFCA, unless an urgent clinical need arose. Both groups discontinued Class I and III AADs at the end of the blanking period (i.e. 3 months after the index procedure).

Patients were seen at out-patient clinics 3, 6, and 12 months after the index procedure and every 6 months after that. The database was locked, and data was analysed when the last included patient completed a 12-month follow-up.

### Outcomes

The **primary efficacy endpoint** was the first recurrence of AF/AT lasting >6 min during the post-blanking follow-up period starting three months after the index procedure or failure to discontinue Class I or III AADs at the beginning of the post-blanking period. The absence or presence of AF/AT on ICM recordings was assessed regularly in a central core lab by experienced cardiologists who were blinded to the treatment allocation of the trial participants.

The **primary clinical endpoint** was a composite of hospitalization for AF/AT, worsening of heart failure, cardioembolic events, or major bleeding. The assessment of this endpoint started on the day of randomization after cardiac surgery in both study groups.

**Secondary outcome measures** included (1) AF/AT burden, (i) within the first year following the index procedure and (ii) throughout the entire duration of the trial, (2) the number of emergency visits due to AF/AT recurrence, worsening of heart failure or other cardiovascular diseases, (3) the number of electrical or pharmacological cardioversions, and (4) RFCA-related complications. The assessment period was identical to that of the primary clinical endpoint.

### Sample size consideration and statistical analysis

The central hypothesis was that the hybrid approach would outperform the surgical ablation in effectiveness. In our previous non-randomized study on concomitant CryoMaze, followed by an electrophysiological study and radiofrequency catheter ablation (RFCA), we found that the AF/tachycardia (AT) recurrence rate was 14% after one year, as determined by 7-day ECG Holter monitoring.^13^ Before catheter ablation (2–3 months after the CryoMaze procedure), any AT/AF (paroxysmal or persistent) was documented in 54% of patients. Therefore, using the more rigorous ECG follow-up based on ICM, we hypothesized that the AF/AT recurrence rate might be as high as 30% in the HG (worst-case scenario) and likely be at least 50% in the SG (the optimistic scenario). Hence, for statistical power analysis, we set the off-antiarrhythmic-drug AF/AT recurrence rate (primary efficacy endpoint) after the hybrid vs. surgical-only ablation to be 30 vs. 50%, respectively. To ensure 90% power of the study to detect the difference between both groups at the 5% significance level (one-sided Z-test), a total of 101 patients had to be analysed in each arm of the trial. Considering a dropout rate of 10%, the trial was designed with an enrolment target of 222 patients. No interim analyses were performed throughout the trial duration.

The assessment of primary and secondary clinical endpoint outcomes in both groups started at randomization, adhering strictly to the intention-to-treat (ITT) principle. This principle was also applied to patients from the HG (i) who refused the RFCA but remained in the trial, (ii) in whom the RFCA procedure was prematurely aborted, and (iii) to patients from the SG in whom the bail-out RFCA was ultimately performed for intractable AF/AT.

A modified ITT (mITT) principle was applied to assess the primary efficacy endpoint. First, a 3-month blanking period was used after the index procedure (i.e. RFCA in the HG and CryoMaze in the SG, respectively), during which no arrhythmic events were counted, and any treatment was allowed to maintain normal sinus rhythm (i.e. electrical or pharmacological cardioversion, AADs initiation, or dose escalation), except repeated RFCA, unless an urgent clinical need arose. Such an approach with a 3-month blanking period has been routinely used in almost all previous AF catheter ablation trials. Second, patients in the HG who died after randomization but before the scheduled date of RFCA were also excluded from the final efficacy analysis. This is because they died following the cardiac surgery procedure during the blanking period when arrhythmic events were not considered anyway.

A per-protocol analysis was carried out subsequently, in which patients from the HG who died before the scheduled RFCA or refused to undergo the procedure were excluded, and patients from the SG scheduled for RFCA for intractable AF/AT were censored at the time of ablation.

Continuous variables are reported as means with standard deviation (SD) or medians with interquartile range (IQR) and compared between the trial groups by a *t*-test for independent samples or Mann–Whitney test, as appropriate. Categorical variables are reported as frequencies and proportions and compared between the trial groups by the chi-square test. Fine–Gray cause-specific cumulative incidence as a function of follow-up time for the primary efficacy endpoint (AF/AT recurrence) and the primary composite clinical endpoint in the presence of competing risk of all-cause death are plotted for the two trial groups.^16^ Also, hazard ratios (HR) are calculated with associated 95% confidence intervals (CI), and Wald tests of the treatment group effect derived from the Fine–Gray subdistribution hazard model.^17,18^ Forest plots derived from the Cox regression model are used for subgroup analyses of primary outcomes and analyses of secondary outcomes. Relative risk (RR) with associated 95% CI and chi-square test is used to compare the incidence of persistent AF/AT between the two groups. In all analyses, the null hypothesis (no difference between treatment groups) was rejected by a two-sided test at the significance level of 0.05. The Cis and *P*-values in the secondary analyses are not adjusted for multiple comparisons. We conducted the statistics using software R, version 4.3.1, and SAS, version 9.3 (SAS Institute, Cary, North Carolina, USA). A second statistician reproduced the primary analysis using SAS Software, version 9.3 (SAS Institute, Cary, North Carolina, USA).

## Results

**Figure 1:**
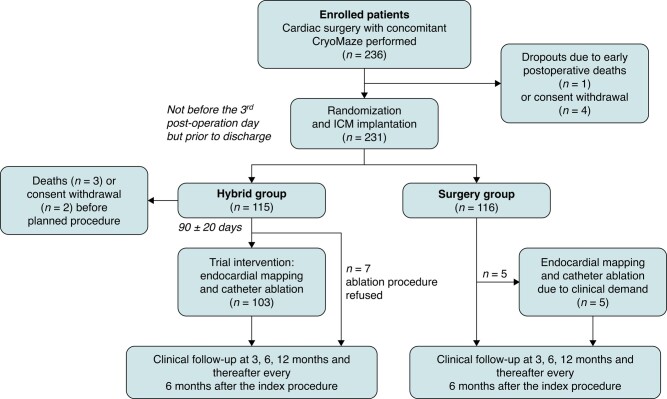
CONSORT diagram of the SURHYB trial. RFCA in the HG and CryoMaze in the SG were considered INDEX procedures. ICM, implantable cardiac monitor.

A total of 236 patients were enrolled at seven sites between 1 May 2019 and 31 March 2022. The CONSORT flowchart of the progress through the phases of the SURHYB trial is shown in *Figure 1*. Due to early postoperative deaths or informed consent withdrawal, 115 and 116 patients were finally assigned to the Hybrid and Surgery Groups, respectively. In the HG, two patients withdrew their consent to the trial before the scheduled RFCA. These patients were excluded, so 113 and 116 patients finally entered the ITT analysis of the primary and secondary clinical endpoints. Three patients in the HG died before the scheduled RFCA (82 ± 27 days after the cardiac surgery procedure), so only 110 and 116 patients in the Hybrid and Surgery Groups, respectively, entered the mITT analysis of the primary efficacy endpoint.

The cross-over rates were relatively modest. Seven patients from the HG refused to undergo RFCA, but they continued in the trial follow-up. Five patients from the SG underwent RFCA (7.3 ± 1.8 months after the index procedure) because of the recurrence of intolerable AF/AT resistant to a rhythm or rate control pharmacological therapy.

Baseline demographic and clinical characteristics were well-balanced between trial groups (*Table 1*). Additional clinical features are provided in Supplementary material online, *Table S1*. Patients had a mean age of 69 years; 69% were males, and 55% had long-standing persistent AF. The mean continuous AF duration before inclusion in the trial was 2.3 ± 2.8 years. Current or prior ineffective use of Class IC or Class III AADs was documented in 90% of patients. Procedural aspects of the CryoMaze and RFCA are shown in Supplementary material online, *Tables S2* and *S3*, and surgical complications are summarized in Supplementary material online, *Table S4*.

**Table 1:** Trial population—baseline clinical characteristics

	Hybrid Group *n* = 113	Surgery Group *n* = 116	Total population *n* = 229	*P* value
Age (years)	68.5 ± 7.2	68.6 ± 7.1	68.5 ± 7.4	0.97
Male	80 (70.8)	79 (68.1)	159 (69.4)	0.78
Body mass index (kg/m^2^)	30.8 ± 4.9	31.2 ± 5.3	31.0 ± 5.1	0.56
Persistent AF	57 (50.4)	47 (40.5)	104 (45.4)	0.18
Long-standing AF	56 (49.6)	69 (59.5)	125 (54.6)	0.18
Congestive heart failure	90 (79.6)	91 (78.4)	181 (79.0)	0.74
NYHA Class				
I	10 (8.8)	10 (8.6)	20 (8.7)	0.95
II	44 (38.9)	36 (31.0)	80 (34.9)	0.21
III	34 (30.1)	44 (37.9)	78 (34.1)	0.33
IV	2 (1.8)	1 (0.9)	3 (1.3)	0.61
CHA_2_DS_2_-VASc score				
0–2	19 (16.8)	21 (18.1)	40 (17.5)	0.80
3–5	78 (69.0)	80 (69.0)	158 (69.0)	0.99
6–9	16 (14.2)	15 (12.9)	31 (13.5)	0.79
Left atrium diameter (cm)	4.8 ± 0.5	4.8 ± 0.5	4.8 ± 0.5	0.76
Left ventricular ejection fraction (%)	56.9 ± 11.5	54.6 ± 10.1	55.8 ± 10.8	0.12
History of electrical cardioversion	48 (42.5)	50 (43.1)	98 (42.8)	0.92
Arterial hypertension	96 (85.0)	105 (90.5)	201 (88.9)	0.23
Diabetes mellitus	40 (35.4)	43 (37.1)	83 (36.2)	0.68
Coronary artery disease	49 (43.4)	50 (43.1)	99 (43.2)	0.97
Transient ischaemic attack/Stroke	14 (12.4)	12 (10.3)	26 (11.4)	0.68

Values are the number (percentage) of patients or mean ± SD. *P-*values refer to the comparison of the Hybrid and Surgery groups.

### Primary efficacy endpoint

**Figure 2:**
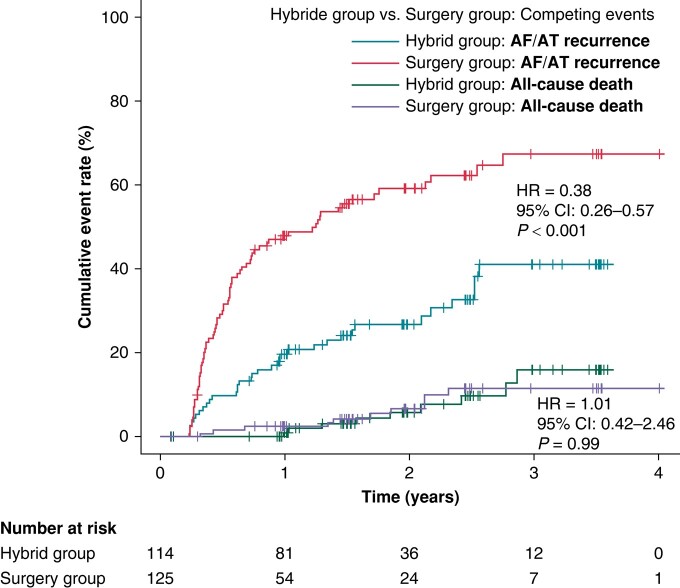
The cumulative rates for the primary efficacy endpoint (AF/AT >6 min). Fine–Gray curves are plotted for SG and HG. All-cause death was used as a competing risk event to assess the difference between the SG and the HG. HR, hazard ratio; CI, confidence interval.

**Figure 3:**
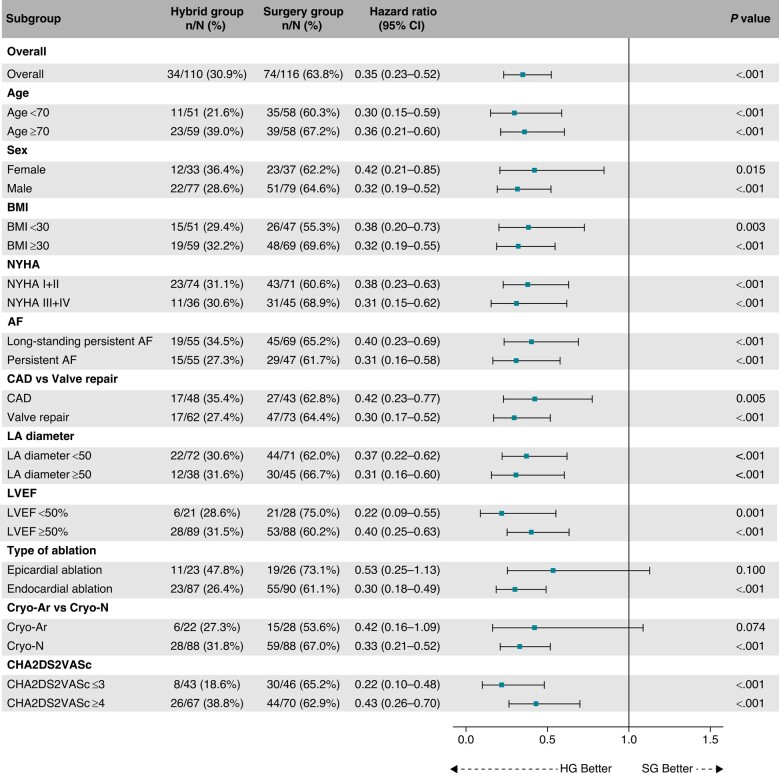
Subgroup analysis for the primary efficacy endpoint. The forest plot derived from Cox regression analysis shows HR estimates (squares) with 95% CI (horizontal bars) for the treatment effects (HG vs. SG) in prespecified subgroups. The widths of the CI and *P*-values are not adjusted for multiple comparisons. No apparent heterogeneity of effects across subgroups using treatment-by-covariate terms was observed. AF, atrial fibrillation; BMI, body mass index; CAD, coronary artery disease; CI, confidence interval; Cryo-Ar, argon-based cryoablation; Cryo-N, nitrogen-based cryoablation; HG, hybrid group; LA, left atrium; LVEF, left ventricular ejection fraction; NYHA, New York Heart Association classification of heart failure; SG, surgery group.

During the median follow-up of 715 days (IQR: 528–1072 days), the recurrence of AF/AT lasting >6 min was significantly reduced in the HG compared to the SG (41.1% vs. 67.4%, HR = 0.38, 95% CI: 0.26–0.57, *P* < 0.001, *Figure 2*). The reduction was consistent over all predefined subgroups of patients (*Figure 3*). Per-protocol analysis also provided a significant risk reduction of AF/AT recurrence in favour of the HG (41.5% vs. 67.5%, HR = 0.39, 95% CI: 0.26–0.59, *P* < 0.001, Supplementary material online, *Figure S2*) as well as analogous consistency across the predefined subgroups of patients (see Supplementary material online, *Figure S3*).

### Primary clinical endpoint

**Figure 4:**
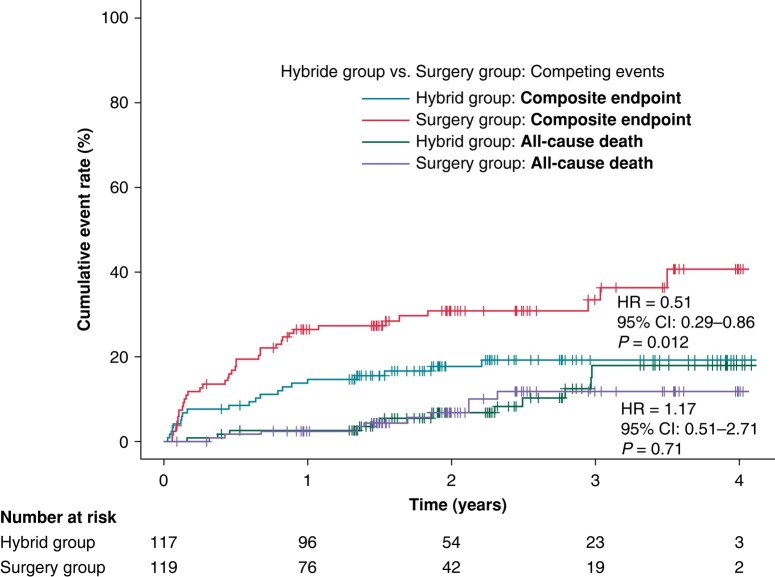
The cumulative rates for the primary clinical endpoint (a composite endpoint of hospitalization for AF/AT recurrence, worsening of heart failure, cardioembolic event, or major bleeding). Legend and the abbreviations are the same as in *Figure 2*.

**Figure 5:**
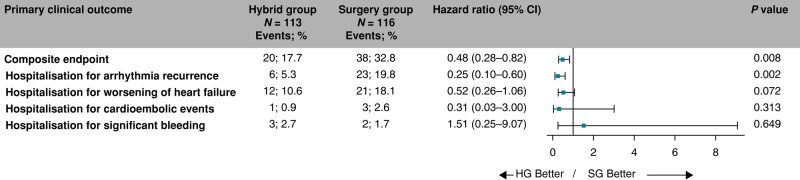
Decomposition of the primary clinical endpoint. The forest plot derived from Cox regression analysis shows HR estimates (squares) with 95% CI (horizontal bars) for the treatment effects (HG vs. SG) for the composite primary clinical endpoint and individual components of the primary clinical endpoint. Data are not adjusted for multiple comparisons. Absolute and relative frequencies of the respective events are also shown. CI, confidence interval; HG, hybrid group; SG, surgery group.

**Figure 6:**
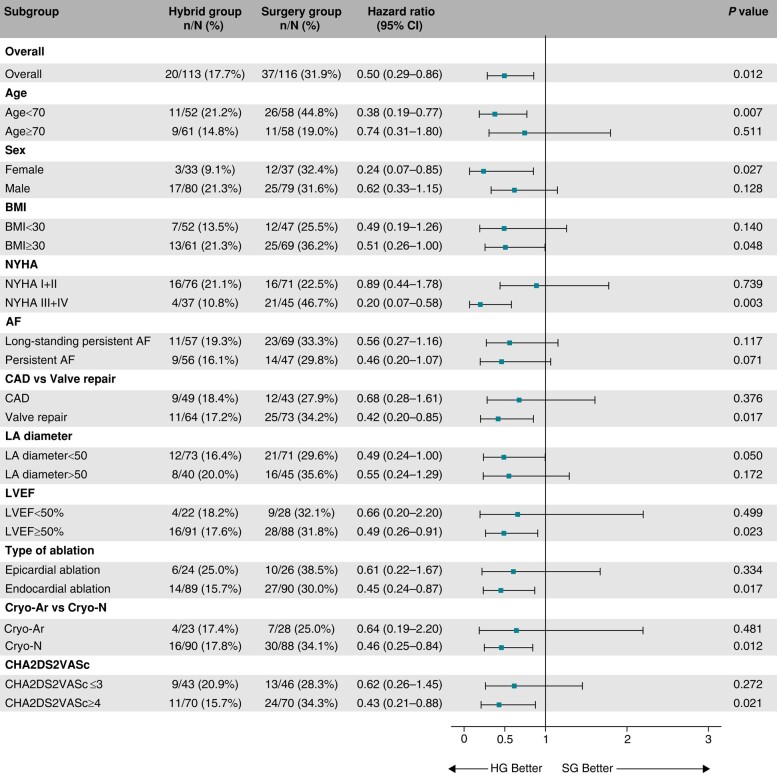
Subgroup analysis for the primary clinical endpoint. The forest plot derived from Cox regression analysis shows HR estimates (squares) with 95% CI (horizontal bars) for the treatment effects (HG vs. SG) in prespecified subgroups. The widths of the CI and *P*-values are not adjusted for multiple comparisons. AF, atrial fibrillation; BMI, body mass index; CAD, coronary artery disease; CI, confidence interval; Cryo-Ar, argon-based cryoablation; Cryo-N, nitrogen-based cryoablation; HG, hybrid group; LA, left atrium; LVEF, left ventricular ejection fraction; NYHA, New York Heart Association classification of heart failure; SG, surgery group.

The occurrence of composite clinical endpoint was significantly lower in the HG compared to the SG (19.9% vs. 40.1%, HR = 0.51, 95% CI: 0.29–0.86, *P* = 0.012, *Figure 4*). Competing all-cause deaths occurred in 12 (10.6%) participants in the HG and 10 (8.6%) SG participants with no significant difference in all-cause mortality between trial groups (HR = 1.17, 95% CI: 0.51–2.71, *P* = 0.71). The estimated HRs for all components of the primary clinical endpoints are shown in *Figure 5*. The overall hazard reduction was mainly driven by hospitalizations for AF/AT recurrence and acute heart failure. The effect of hybrid ablation was consistently seen across the predefined subgroups of patients (*Figure 6*).

Per-protocol analysis also demonstrated risk reduction for the occurrence of composite clinical endpoint in the HG (20.1% vs. 40.2%, HR = 0.53, 95% CI: 0.31–0.90, *P* = 0.02, see Supplementary material online, *Figure S4*). Like in ITT analysis, the overall hazard reduction was mainly driven by hospitalizations for AF/AT recurrence and acute heart failure (see Supplementary material online, *Figure S5*). The consistent effect of hybrid ablation across the predefined subgroups of patients as per-protocol analysis is shown in Supplementary material online, *Figure S6*.

### Secondary outcomes

Significantly fewer patients in the HG suffered from persistent AF/AT following the index procedure [7 (6.2%) vs. 31 (26.7%), RR = 0.24, 95% CI: 0.11–0.52, *P* < 0.001]. In 70 patients (27 patients in the HG and 43 patients in the SG) who converted from original persistent to paroxysmal AF/AT after the index procedure, AF/AT burden (expressed in % of the time) was significantly lower in the HG compared to the SG either within the first year (median 0.0%, IQR: 0.0–0.9% vs. median 0.25%, IQR: 0.0–4.1%, *P* = 0.005) or throughout the entire duration of the trial (median 0.1%, IQR: 0–2.8% vs. median 1.9%, IQR: 0.1–23.6%, *P* < 0.001). The longest detected AF/AT episode was also significantly shorter in the HG compared to the SG either within the first year (median: 0 min, IQR: 0–0 min, mean ± SD: 36 ± 106 min vs. median: 0 min, IQR: 0–84 min, mean ± SD: 149 ± 433 min, *P* < 0.001) or throughout the entire duration of the trial (median: 0 min, IQR: 0–45 min, mean ± SD: 110 ± 392 min vs. median: 69 min, IQR: 0–242 min, mean ± SD: 318 ± 680 min, *P* < 0.001).

**Figure 7:**
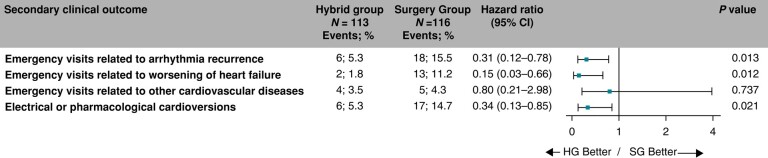
Hazard ratios for the secondary clinical endpoints. The forest plot derived from Cox regression analysis shows HR estimates (squares) with 95% CI (horizontal bars) for the treatment effect (HG vs. SG) for individual secondary clinical endpoints. Data are not adjusted for multiple comparisons. Absolute and relative frequencies of the respective events are also shown. CI, confidence interval; HG, hybrid group; SG, surgery group.

The risks for an emergency visit for AF/AT recurrence, for worsening of heart failure, and risk for pharmacological or electrical cardioversion, respectively, were significantly reduced (5.3% vs. 15.5%, HR = 0.31, 95% CI: 0.12–0.78, *P* = 0.013; 1.8% vs. 11.2%, HR = 0.15, 95% CI: 0.03–0.66, *P* = 0.012; and 5.3% vs. 14.7%, HR = 0.34, 95% CI: 0.13–0.85, *P* = 0.021, respectively), in the Hybrid compared to the SG (*Figure 7*). Comparable statistically significant reductions in emergency visits for AF/AT recurrence, worsening of heart failure, and pharmacological or electrical cardioversion were demonstrated in the per-protocol analysis (see Supplementary material online, *Figure S7*).

Major procedural complications, defined as those that result in permanent injury or death, require intervention, or prolong or require hospitalization, were present in 2 (1.9%) patients after RFCA. One femoral pseudoaneurysm was treated by thrombin injection without sequelae, and the case of haematuria prolonging the hospitalization was treated conservatively. There were 4 (3.9%) minor procedural complications related to RFCA. Details are provided in Supplementary material online, *Table S5*.

## Discussion

In this multicentre, randomized trial in structural heart disease patients with non-paroxysmal AF, we found that a hybrid ablation strategy for AF, i.e. catheter ablation performed three months after concomitant CryoMaze, was effective and safe. It resulted in higher freedom from AF/AT and improved clinical outcomes, primarily driven by reduced hospitalizations due to arrhythmia recurrence and heart failure.

More than a quarter of patients amenable to cardiac surgery suffer from AF.^19^ Current evidence-based recommendations support surgical ablation in patients with AF undergoing cardiac surgery for other indications.^15,20^ According to the 2017 guidelines of the Society of Thoracic Surgeons,^21^ surgical ablation is highly recommended as a Class IA procedure to restore sinus rhythm along with mitral valve surgery. It is also recommended as a Class IB procedure in conjunction with coronary artery bypass, aortic valve replacement, or coronary artery bypass with aortic valve replacement.

Recent reports on concomitant CryoMaze procedures showed efficacy rates between 47 and 76%, depending on the intensity of rhythm monitoring.^10,22,23^ The only two small observational studies on hybrid procedures (with RFCA after the concomitant CryoMaze procedure) reported overall freedom from AF/AT to be 86% at 12 months^13^ or 73% after a 10-year follow-up,^24^ with the former using regular 7-day ECG monitoring, and the latter only 24-h ECG monitoring. Both studies documented a considerable proportion of incomplete lesion schemes after CryoMaze. The SURHYB trial, the first randomized trial on this topic, showed an impressive reduction in recurrent AF/AT after the RFCA, which reached 62%. Moreover, ECG monitoring was done very precisely using an ICM, and the effect of RFCA was consistent across the multiple predefined subgroups of patients.

There are several reasons why hybrid procedures are generally more effective than surgical ablation, whether performed epicardially or endocardially, using cryoenergy^13,24^ or RF energy for the primary surgical lesion set.^25–27^ In the first place, the hybrid approach provides more durable, transmural lesions because of the combined use of epicardial and endocardial ablation. In addition, a sequential treatment strategy that considerably limits the net recoverability of ablated myocardium is crucial in controlling both PVI as well as macroreentrant Ats.^28^ CTI-dependent flutter, one of the most often regular arrhythmias in patients after surgical AF ablation, can also be easily prevented by RFCA. During RFCA, it is also possible to target multiple residual microreentrant ATs, which could not be treated by anatomically guided CryoMaze procedure and can be present only after some distance since the surgical procedure due to the gradual maturation of lesions. Recently, three randomized studies have confirmed the effectiveness of hybrid therapy for AF. The comparator was either RFCA alone,^29^ repeated RFCA alone,^30^ or surgical ablation alone.^31^ It should be noted, however, that these studies differed significantly from our study. They focused on a population with primary electrical disease and without structural heart disease. As such, the surgical ablation consisted of a standalone minimally invasive surgical approach and not a surgical ablation being performed as a concomitant procedure during surgery for structural heart disease. Additionally, none of these studies investigated the clinical outcomes, so the SURHYB is the first to provide reliable data on the clinical benefit of hybrid ablation for AF in the mid-term perspective.

The reduction in AF/AT burden in the SURHYB trial was associated with a decrease in emergency visits, cardioversions, and hospital admissions not only due to AF/AT recurrence but also because of the worsening of heart failure, as these two conditions are closely related. In CABANA (*Catheter Aablation Versus Anti-arrhythmic Drug Therapy for Atrial Fibrillation*), a trial that compared invasive and medical treatment for AF, the significant drop in arrhythmia burden in patients who underwent the catheter ablation was associated with a substantial reduction in the combined endpoint of death or cardiovascular hospitalization by 17%.^32^ As assessed from CABANA Supplementary material online, the percentage of hospitalizations for any of AF/AT, heart failure, stroke/transitory ichemic attack, or serious bleeding, i.e. combination comparable to composite clinical endpoint in SURHYB, was 18 vs. 21% in ablation vs. control group, thus with relative reduction by 13% only. Such effect is substantially smaller than that in the HG of SURHYB trial (reduction by 49%) even if the risk reduction of AF/AT recurrence was only slightly smaller in CABANA vs. SURHYB (by 48 vs. 62%, respectively). Higher clinical benefit in the SURHYB trial might be present since the RFCA in the SURHYB trials was already the second intervention for AF, and the lesion completeness could be higher compared to a single intervention in CABANA. Even more importantly, a different study population may explain a more profound effect of the hybrid strategy on the clinical events in the SURHYB trial. Unlike CABANA, the SURHYB trial included patients with advanced structural heart disease, multiple comorbidities, and only non-paroxysmal AF, in whom AF/AT recurrences might more easily trigger various cardiovascular events, as it was previously shown in the CASTLE trial,^33^ and recently in the CASTLE-HTx trial.^34^

RFCA procedure in the SURHYB trial was associated with a low risk of complications (and zero rates of those with long-term sequelae), reflecting the improved safety of contemporary RFCA. Therefore, the common risks associated with cardiac surgery and concomitant CryoMaze were not significantly increased by adding RFCA in this trial. However, this was achieved in a relatively small cohort of patients who were treated in high-volume cardiovascular centres by experienced operators using ultrasound-navigated groin puncture and intracardiac echocardiography to navigate transseptal puncture, catheter maneuvering, and instantaneous monitoring of potential complications. Such outcomes in terms of safety may not apply to real-world procedures in low-volume and less experienced centres.

The trial has limitations that need to be addressed. First, the open-label design might bias the rate of the primary clinical events even if this was minimized by independent endpoint adjudication. Second, there was a delay between the randomization and index procedure in the HG and, consequently, a 3-month difference in follow-up time between the trial groups regarding AF/AT recurrence. This could not be avoided, as postponing RFCA in sequential hybrid procedures is necessary to heal and consolidate surgical lesions properly. For all clinical outcomes, however, no blanking period was used, and all events were counted strictly from the time of randomization following the ITT protocol. Third, the evaluation of the ICM recordings might be compromised by the risk of arrhythmia underdetection. Overdetection may be almost excluded since all ICM arrhythmia recordings automatically evaluated as AF or high ventricular rate were manually overseen. Yet, we cannot exclude mistakenly assessed recordings, but these were likely rare and balanced between the trial groups. AF/AT recurrence was defined as an episode of AF/AT lasting >6 min because shorter AF/AT episodes (those lasting >30 s but <6 min) could have been missed due to principles of the built-in detection algorithms of ICM with different sensitivities for AF detection.

## Conclusion

In a cohort of patients with non-paroxysmal AF undergoing cardiac surgery for structural heart disease and CryoMaze, catheter ablation performed sequentially after the surgical procedure resulted in higher freedom from AF/AT and reduction of hospitalization for arrhythmia recurrence and acute heart failure when compared to CryoMaze alone. Such pre-emptively performed catheter ablation after the CryoMaze procedure reduced emergency out-patient visits for cardiovascular reasons and decreased the need for electrical or pharmacological cardioversions of recurrent AF/AT. This was achieved with minimal risk of procedure-related complications. The clinical outcomes of enrolled patients are still being monitored as part of the trial extension.

## Supplementary material

Supplementary material is available at *Europace* online.

## Supplementary Material

euae040_Supplementary_Data

## Data Availability

The data underlying this article will be shared on reasonable request to the corresponding author.
