# Supplementary material for: Sequential hybrid ablation vs. surgical CryoMaze alone for treatment of atrial fibrillation: results of multicentre randomized controlled trial
Source: Europace. 2024 Feb 2;26(2):euae040. doi: 10.1093/europace/euae040 (PMC10872694; doi:10.1093/europace/euae040)
Supplement: euae040_Supplementary_Data [file euae040_supplementary_data.zip › Supplementary Appendix Surhyb Europace_final.docx]

**Supplementary Appendix**

Supplement to: Bulava A, Wichterle D, Mokráček A, et al. Sequential Hybrid Ablation versus Surgical CryoMaze Alone for Treatment of Atrial Fibrillation: Results of Multicentre Randomized Controlled Trial. Europace 2024; XXX. DOI: XXX

The authors have provided this appendix to give readers additional information about the work.

**Table of Contents**

List of Investigators ....................................................................................................................3

Trial Organization ………………………………………………………………………………………………………………….4

Supplemental Tables ................................................................................................................. 6

Table S1. Baseline characteristics – addenda ............................................................................ 6

Table S2. Cardiac surgery characteristics ……………………………………………………………………...……… 7

Table S3. Characteristics of the catheter ablation procedure …………........................................ 9

Table S4. Complications of the cardiac surgery procedure ……………………………………………....... 10

Table S5. Complications of the catheter ablation procedure .................................................. 11

Table S6. Mode of death …………………………………………………………………………………………………... 12

Supplemental Figures ............................................................................................................. 13

Figure S1. Scheme of surgical cryolesions ……………………………….……………………………............... 13

Figure S2. The cumulative rates for the primary efficacy endpoint as per-protocol analysis….14

Figure S3. Subgroup analysis for the primary efficacy endpoint as per-protocol analysis ….... 15

Figure S4. The cumulative rates for the primary clinical endpoint (a composite endpoint of hospitalisation for AF/AT recurrence, worsening of heart failure, cardioembolic event, or major bleeding) as per-protocol analysis ……………………………………………………………………….……………. 16

Figure S5. Decomposition of the primary clinical endpoint as per-protocol analysis….…......... 17

Figure S6. Subgroup analysis for the primary clinical endpoint as per-protocol analysis ....... 18

Figure S7. Hazard ratios for secondary clinical endpoints as per-protocol analysis ………….…. 19

Study definition of clinical events requiring hospitalisation…………......................................... 20

Study definition of clinical events not requiring hospitalisation…………................................... 22

**List of Investigators**

Alan Bulava, MD, PhD^1,2^, Aleš Mokráček, MD, PhD^1,2^, Jiří Haniš, MD, PhD^1^, Přemysl Hájek, MD^1^, Iva Šafaříková, PhD^1,2^, David Sitek^1^, Adam Novotný^1^, Pavel Osmančík, MD^3^, PhD, Petr Kačer, MD, PhD^3^, Dalibor Heřman, MD, PhD^3^, Vitalii Rizov, MD^3^, Ondřej Süssenbek, MD^3^, Dan Wichterle, MD, PhD^4^, Petr Budera, MD, PhD^4^, Ondrej Szárszoi, MD, PhD^4^, Lukáš Salavec, MD^4^, Petr Peichl, MD, PhD^4^, Josef Kautzner, MD, PhD^4^, Ivan Netuka, MD, PhD^4^, Jiří Ondrášek, MD, PhD^5^, Linda Vetešková, MD^5^, Petr Němec, MD, PhD^5^, Jana Frantová^5^, Tomáš Skála, MD, PhD^6^, Petr Šantavý, MD, PhD^6^, Dalibor Klimeš^6^, Mariwan Majid, MD^7^, Miroslav Kolesár, MD, PhD^7^, Pavel Červinka, MD, PhD^7^, Jan Chovančík, MD, PhD^8^, Piotr Branny, MD^8^, Otakar Jiravský, MD^8^

Affiliations:

1 Cardiac Centre, České Budějovice Hospital, České Budějovice, Czechia

2 Faculty of Health and Social Sciences, University of South Bohemia in České Budějovice, Czechia

3 3^rd^ Faculty of Medicine, Charles University and University Hospital Královské Vinohrady, Prague, Czechia

4 Institute for Clinical and Experimental Medicine, Prague, Czechia

5 Centre of Cardiovascular Surgery and Transplantation, Brno, Czechia

6 Faculty of Medicine and Dentistry, Palacký University and University Hospital Olomouc, Olomouc, Czechia

7 Masaryk Hospital, Ústí nad Labem, Czechia

8 Hospital Agel Třinec - Podlesí, Třinec, Czechia

9 Faculty of Biomedical Engineering, Czech Technical University in Prague, Kladno, Czechia

Authors contribution:

Data analysis was performed by a trial statistician (MR). AB wrote the first draft of the manuscript supported by DW and PO. Members of the Trial Steering Committee and all Local Data Managers ensured the data's accuracy and completeness and the trial's fidelity to the protocol. All authors contributed to the review and revision of the manuscript.

**Trial Organization**

Trial Steering Committee

Alan Bulava, MD, PhD^1^, Pavel Osmančík, MD, PhD^2^, Dan Wichterle, MD, PhD^3^

Data Safety Monitoring Board

Adrian Bystrianský, MD, PhD^4^, Ondřej Toman, MD, PhD^5^, Jiří Plášek, MD, PhD^6^

Endpoint Review Committee

Jan Kozák, MD, PhD^5^, Ondřej Toman, MD, PhD^5^, Jiří Plášek, MD, PhD^6^

Central Holter Assessment

David Sitek^1^, Adam Novotný^1^

Local Data Managers

Jiří Haniš, MD^1^, Pavel Osmančík, MD, PhD^2^, Dan Wichterle, MD, PhD^3^, Linda Vetešková, MD^7^, Tomáš Skála, MD, PhD^8^, Vitalii Rizov, MD^9^, Jan Chovančík, MD, PhD^10^

Statistical Design and Analysis

Marian Rybář^11^, Radka Štěpánová^12^, Adam Svobodník^12^

Affiliations:

1 Cardiac Centre, České Budějovice Hospital, České Budějovice, Czechia

2 3^rd^ Faculty of Medicine, Charles University and University Hospital Královské Vinohrady, Prague, Czechia

3 Institute for Clinical and Experimental Medicine, Prague, Czechia

4 Middle Slovak Institute of Cardiovascular Diseases, Banská Bystrica, Slovakia

5 University Hospital Brno - Bohunice, Brno, Czechia

6 University Hospital Ostrava, Ostrava, Czechia

7 Centre of Cardiovascular Surgery and Transplantation, Brno, Czechia

8 Faculty of Medicine and Dentistry, Palacký University and University Hospital Olomouc, Olomouc, Czechia

9 Masaryk Hospital, Ústí nad Labem, Czechia

10 Hospital Agel Třinec - Podlesí, Třinec, Czechia

11 Faculty of Biomedical Engineering, Czech Technical University in Prague, Kladno, Czechia

12 Faculty of Medicine, Masaryk University, Brno, Czechia

**Supplemental Tables**

**Table S1. Baseline characteristics – addenda**

|  | **Hybrid Group n = 113** | **Surgery Group n = 116** | **Total population n = 229** |
| --- | --- | --- | --- |
| **LA dimensions** |  |  |  |
| LA size – short axis (cm) | 5.3 ± 0.8 | 5.2 ± 0.6 | 5.2 ± 0.9 |
| LA size – long axis (cm) | 6.4 ± 1.3 | 6.7 ± 1.2 | 6.6 ± 1.2 |
| LA area (cm^2^) | 35.4 ± 17.6 | 37.4 ± 23.8 | 36.5 ± 21.0 |
| LAVI (ml/m^2^) | 54.3 ± 21.6 | 56.1 ± 27.1 | 55.2 ± 24.5 |
| **Laboratory examinations** |  |  |  |
| NT-proBNP (ng/l) | 1945 ± 1730 | 1974 ± 1963 | 1960 ± 1849 |
| Urea (mmol/l) | 7.3 ± 3.8 | 6.9 ± 2.6 | 7.1 ± 3.2 |
| Creatinine (μmol/l) | 98.3 ± 30.3 | 92.6 ± 20.4 | 95.3 ± 25.7 |
| CRP (mg/l) | 5.9 ± 12.4 | 5.4 ± 11.3 | 5.6 ± 11.8 |
| ALT (μkat/l) | 0.6 ± 0.4 | 0.6 ± 0.5 | 0.6 ± 0.4 |
| AST (μkat/l) | 0.5 ± 0.2 | 0.5 ± 0.3 | 0.5 ± 0.2 |
| Haemoglobin (g/l) | 142 ± 14 | 141 ± 16 | 142 ± 16 |
| **Antiarrhythmic drugs at admission** |  |  |  |
| Amiodarone | 31 (27.4) | 38 (32.8) | 69 (30.1) |
| Propafenone | 15 (13.3) | 9 (7.8) | 24 (10.5) |
| Sotalol | 5 (4.4) | 6 (5.2) | 11 (4.8) |
| Flecainide | 0 (0) | 0 (0) | 0 (0) |
| Dronedarone | 0 (0) | 0 (0) | 0 (0) |
| Beta-blockers | 34 (30.1) | 32 (27.6) | 66 (28.8) |
| Others | 0 (0) | 0 (0) | 0 (0) |
| **Anticoagulation therapy on admission** |  |  |  |
| NOAC | 49 (43.4) | 42 (36.2) | 91 (39.7) |
| Warfarin | 44 (38.9) | 57 (49.1) | 101 (44.1) |
| None | 20 (17.7) | 17 (14.7) | 37 (16.2) |

Values are the number (percentage) of patients or mean ± standard deviation. No statistically significant differences were noted between the groups. ALT, Alanine transaminase; AST, Aspartate transaminase; CRP, C-reactive protein; LA, left atrial; LAVI, left atrial volume index; NOAC, novel oral anticoagulant; NT-proBNP, N-terminal pro-brain natriuretic peptide.

**Table S2. Cardiac surgery characteristics**

|  | **Hybrid Group n = 113** | **Surgery Group n = 116** | **Total population n = 229** |
| --- | --- | --- | --- |
| Procedural time (min) | 223 ± 60 | 233 ± 67 | 228 ± 64 |
| Cardiopulmonary bypass time (min) | 121 ± 41 | 124 ± 36 | 123 ± 38 |
| Aortic clamp time (min) | 86 ± 36 | 90 ± 33 | 88 ± 35 |
| Hospitalisation length (nights) | 13.6 ± 7.5 | 13.1 ± 6.0 | 13.3 ± 6.7 |
| **Type of procedure** |  |  |  |
| Coronary artery bypass grafting | 50 (44.2) | 42 (36.2) | 92 (40.2) |
| Number of bypass grafts | 2.2 ± 1.0 | 2.2 ± 1.0 | 2.2 ± 1.0 |
| Complete revascularisation^*^ | 42 (84.0) | 33 (78.6) | 75 (81.5) |
| Mitral valve repair | 28 (24.8) | 30 (25.9) | 58 (25.3) |
| Mitral valve replacement | 15 (13.3) | 13 (11.2) | 28 (12.2) |
| Tricuspid valve repair | 25 (22.1) | 20 (17.2) | 45 (19.7) |
| Aortic valve replacement | 40 (35.4) | 42 (36.2) | 82 (35.8) |
| Endocardial cryoablation | 88 (77.9) | 90 (77.6) | 178 (77.7) |
| Epicardial cryoablation | 25 (21.1) | 26 (22.4) | 51 (22.3) |
| **Type of cryoenergy** |  |  |  |
| Argon-based cryoablation | 23 (20.4) | 28 (24.1) | 51 (22.3) |
| Nitrogen-based cryoablation | 90 (79.6) | 88 (75.9) | 178 (77.7) |
| **CryoMaze ablation details** |  |  |  |
| Left pulmonary veins ablated | 112 (99.1) | 115 (99.1) | 227 (99.1) |
| Right pulmonary veins ablated | 113 (100.0) | 115 (99.1) | 228 (99.6) |
| Left atrial appendage occlusion | 101 (89.4) | 103 (88.8) | 204 (89.1) |
| AtriClip device | 69 (61.1) | 69 (59.5) | 138 (60.3) |
| Staplers | 0 (0) | 1 (0.9) | 1 (0.4) |
| Cut-and-sew technique | 32 (28.3) | 33 (28.4) | 65 (28.4) |
| Left atrial box lesion created | 113 (100.0) | 114 (98.3) | 227 (99.1) |
| Mitral isthmus line created | 97 (85.8) | 105 (90.5) | 202 (88.2) |
| Marshall ligament destruction | 81 (71.7) | 81 (69.8) | 162 (70.7) |
| Superior vena cava ablated | 13 (11.5) | 11 (9.5) | 24 (10.5) |
| Inferior vena cava ablated | 0 (0.0) | 0 (0.0) | 0 (0.0) |
| Intercaval line created | 45 (39.8) | 38 (32.8) | 83 (36.2) |
| Cavotricuspid isthmus line created | 8 (7.1) | 8 (6.9) | 16 (7.0) |
| Other lines in the right atrium | 19 (16.8) | 21 (18.1) | 40 (17.5) |
| Other lines in the left atrium | 0 (0.0) | 6 (5.2) | 6 (2.6) |
| **Rhythm at discharge from the surgery department** | | | |
| Sinus rhythm | 78 (69.0) | 81 (69.8) | 159 (69.4) |
| Atrial tachycardia | 2 (1.8) | 2 (1.7) | 4 (1.7) |
| Atrial fibrillation | 27 (23.9) | 25 (21.6) | 52 (22.7) |
| Typical atrial flutter | 0 (0) | 0 (0) | 0 (0) |
| Junctional rhythm | 1 (0.9) | 0 (0.0) | 1 (0.4) |
| Paced rhythm | 5 (4.4) | 8 (6.9) | 13 (5.7) |

* Percentage of complete revascularisation in the subgroup of patients undergoing bypass surgery

Values are the number (percentage) of patients or mean ± standard deviation. No statistically significant differences were noted between the groups, except for the slightly higher number of other than prespecified left atrial lesions in the Surgery Group (5.2% vs. 0.0%, P = 0.045).

**Table S3. Characteristics of the catheter ablation procedure**

| **Ablation Procedure Data (n = 103)** |  |
| --- | --- |
| Time from CryoMaze to catheter ablation (days) | 105 ± 35 |
| Procedural time (min) | 138 ± 52 |
| Radiofrequency energy application time (min) | 27.0 ± 16.3 |
| Fluoroscopy time (min) | 6.9 ± 3.2 |
| Fluoroscopy dose (mGy.cm^2^) | 6507 ± 7300 |
| Hospitalisation length (nights) | 2.6 ± 1.2 |
| **Ablation procedure details** |  |
| Patients presented in SR | 74 (71.8) |
| Ablation catheter with CFS | 81 (78.6) |
| Ablation catheter w/o CFS | 22 (21.4) |
| **Rhythm at discharge from the cardiology department** | |
| Sinus rhythm | 100 (97.1) |
| Atrial fibrillation | 3 (2.9) |
| Junctional rhythm | 0 (0.0) |
| Paced rhythm | 0 (0.0) |

Values are the number (percentage) of patients or mean ± standard deviation.

Seven patients from the Hybrid Group refused to undergo the electrophysiology mapping and ablation procedure. Data are provided for the remaining 103 patients.

CFS, contact force sensor-equipped ablation catheters; SR, sinus rhythm; RFCA, radiofrequency catheter ablation

**Table S4. Complications of the cardiac surgery procedure**

| **Surgery Complications (n = 229)** | **N** | **%** |
| --- | --- | --- |
| **Periprocedural complications*** | **20** | **8.7** |
| Death | 0 | 0 |
| Acute myocardial infarction | 1 | 0.4 |
| Bleeding requiring immediate intervention | 2 | 0.9 |
| Pneumothorax requiring drainage | 2 | 0.9 |
| Pleural effusion | 4 | 1.7 |
| Acute stroke or transitory ischemic attack | 0 | 0 |
| Atrioventricular block | 9 | 3.9 |
| Sinoatrial block | 2 | 0.9 |
| **Postprocedural complications*** | **93** | **40.6** |
| Death | 0 | 0 |
| Acute stroke or transitory ischemic attack | 3 | 1.3 |
| Phrenic nerve palsy | 2 | 0.9 |
| Atrioventricular block requiring PM implantation | 13 | 5.7 |
| Sinoatrial block requiring PM implantation | 3 | 1.3 |
| Renal failure requiring haemodialysis | 2 | 0.9 |
| Worsening of renal function not requiring haemodialysis | 7 | 3.1 |
| Bleeding requiring surgical intervention | 6 | 2.6 |
| Non-significant bleeding w/o surgical intervention | 2 | 0.9 |
| Pneumothorax requiring drainage | 3 | 1.3 |
| Minor pneumothorax not requiring drainage | 4 | 1.7 |
| Pleural effusion requiring drainage | 16 | 7.0 |
| Pleural effusion not requiring drainage | 19 | 8.3 |
| Respiratory insufficiency requiring prolonged MV | 2 | 0.9 |
| Respiratory insufficiency not requiring MV | 6 | 2.6 |
| Bronchopneumonia requiring antibiotics | 5 | 2.2 |

The data are provided for patients who entered the final analysis (n = 226).

MV, mechanical ventilation; PM, permanent pacemaker

*Multiple complications may have been documented in the same patient

**Table S5. Complications of the catheter ablation procedure**

| **Complications of the Ablation Procedure (n = 103)** | **N** | **%** |
| --- | --- | --- |
| **Periprocedural complications*** | **0** | **0** |
| Death | 0 | 0 |
| Pericardial tamponade requiring pericardiocentesis | 0 | 0 |
| Pericardial effusion not requiring pericardiocentesis | 0 | 0 |
| Stroke or transitory ischemic attack | 0 | 0 |
| Phrenic or vagal nerve injury | 0 | 0 |
| Atrioventricular block | 0 | 0 |
| Sinoatrial block | 0 | 0 |
| Haemorrhage requiring surgical intervention | 0 | 0 |
| **Postprocedural major complications*** | **2** | **1.9** |
| Death | 0 | 0 |
| Pericardial tamponade requiring pericardiocentesis | 0 | 0 |
| Pericardial effusion not requiring pericardiocentesis | 0 | 0 |
| Pericarditis | 0 | 0 |
| Stroke or transitory ischemic attack | 0 | 0 |
| Phrenic or vagal nerve injury | 0 | 0 |
| Atrioventricular block requiring PM implantation | 0 | 0 |
| Sinoatrial block requiring PM implantation | 0 | 0 |
| Haemorrhage requiring surgical intervention | 0 | 0 |
| Symptomatic pulmonary vein stenosis | 0 | 0 |
| Haematuria prolonging hospitalisation | 1 | 1.0 |
| Femoral artery pseudoaneurysm requiring intervention | 1 | 1.0 |
| **Postprocedural minor complications*** | **4** | **3.9** |
| Small arteriovenous fistula at groin ** | 1 | 1.0 |
| Groin hematoma not requiring intervention | 3 | 2.9 |

Two patients from the Hybrid Group withdrew their informed consent, three died before the scheduled procedure, and seven refused to undergo the electrophysiology mapping and ablation procedure. Data are provided for the remaining 103 patients.

PM, permanent pacemaker

* There was not more than one documented event per patient

** Resolved after manual compression

**Table S6. Mode of death**

| Mode of death | Hybrid Group  n = 12 | Surgery Group  n = 10 | Total population  n = 22 |
| --- | --- | --- | --- |
| Acute myocardial infarction | 0 (0%) | 1 (10%) | 1 (5%) |
| Bilateral pneumonia | 1 (8%) | 2 (20%) | 3 (14%) |
| COVID-19 | 1 (8%) | 2 (20%) | 3 (14%) |
| Infective endocarditis | 2 (17%) | 0 (0%) | 2 (9%) |
| Mediastinitis | 1 (8%) | 0 (0%) | 1 (5%) |
| Multiple organ failure | 0 (0%) | 1 (10%) | 1 (5%) |
| Respiratory failure | 0 (0%) | 1 (10%) | 1 (5%) |
| Sepsis of unknown origin | 1 (8%) | 0 (0%) | 1 (5%) |
| Stroke | 0 (0%) | 1 (10%) | 1 (5%) |
| Sudden cardiac death | 2 (17%) | 1 (10%) | 3 (14%) |
| Suicide | 1 (8%) | 0 (0%) | 1 (5%) |
| End-stage heart failure | 3 (25%) | 0 (0%) | 3 (14%) |
| Unknown | 0 (0%) | 1 (10%) | 1 (5%) |

Values are the number (percentage) of patients.

**Supplemental Figures**

**Figure S1. Scheme of surgical cryolesions**


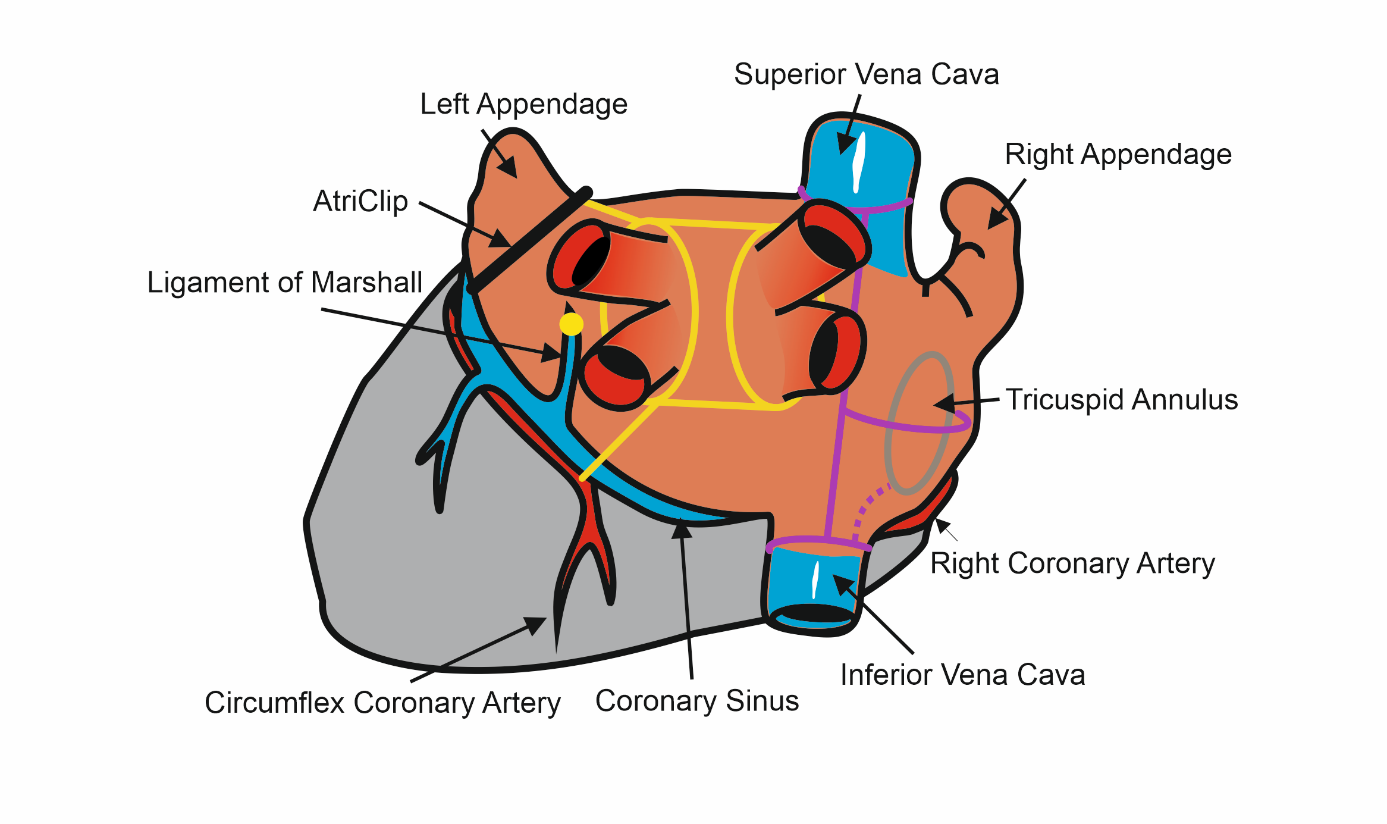


The CryoMaze procedure consisted of mandatory circular lesions (drawn in yellow colour) around the ipsilateral right and left pulmonary veins with linear lesions toward the auricle and linear lesions between the superior and inferior pulmonary veins to isolate the LA posterior wall. A mitral isthmus ablation line was created in all patients from the inferior connecting lesion towards the mitral annulus. In addition, the ligament of Marshall was cut off (yellow dot) and left atrial appendage exclusion was performed using preferably the AtriClip device (black line), but other techniques of exclusion were also allowed (stapler, cut-and-sew). Lin In addition, a connecting line between the left superior pulmonary vein and the base of the left atrial appendage was created. Right atrial lesions (purple) were performed at the surgeon's discretion. Such lesions may have included but were not limited to superior/inferior vena cava isolation, intercaval lesion, lateral line connecting intercaval lesion to the tricuspid annulus, and cavotricuspid isthmus lesion (dotted purple line).

**Figure S2.** **The cumulative rates for the primary efficacy endpoint as per-protocol analysis**





Fine-Grey curves are plotted for the Surgery Group (red) and Hybrid Group (black). All-cause death was used as a competing risk event to assess the difference between the Surgery Group (blue) and the Hybrid Group (green).

HR, hazard ratio; CI, confidence interval

**Figure S3. Subgroup analysis for the primary efficacy endpoint as per-protocol analysis**


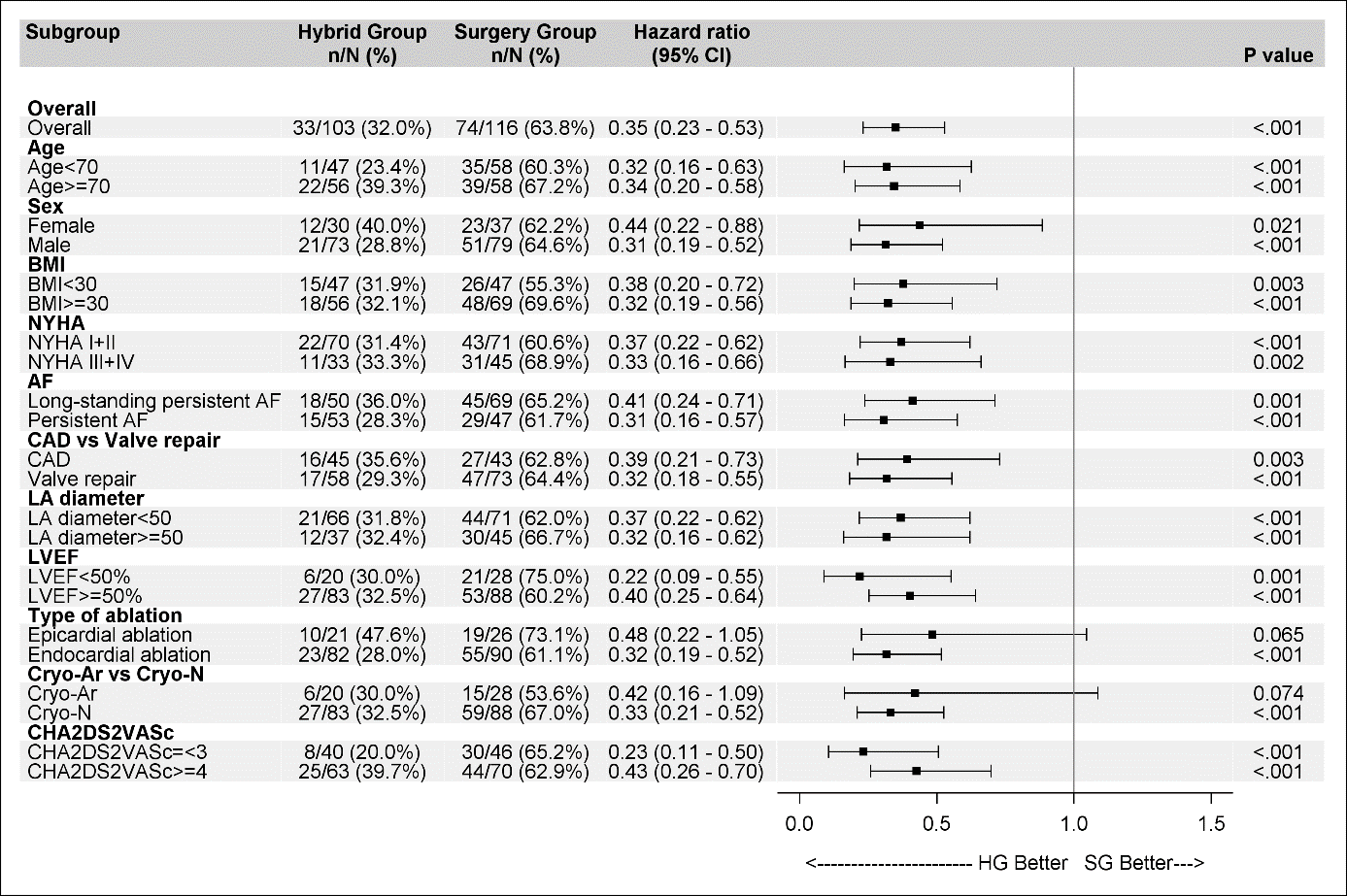


N, number of all patients; n, number of patients with the event

The forest plot derived from Cox regression analysis shows hazard ratio estimates (squares) with 95% CI (horizontal bars) for the treatment effects (Hybrid Group vs. Surgery Group) in prespecified subgroups. The widths of the CI and P-values are not adjusted for multiple comparisons. No apparent heterogeneity of effects across subgroups using treatment-by-covariate terms was observed.

AF, atrial fibrillation; BMI, body mass index; CAD, coronary artery disease; CI, confidence interval; Cryo-Ar, argon-based cryoablation; Cryo-N, nitrogen-based cryoablation; HG, hybrid group; LA, left atrium; LVEF, left ventricular ejection fraction; NYHA, New York Heart Association classification of heart failure; SG, surgery group.

**Figure S4. The cumulative rates for the primary clinical endpoint (a composite endpoint of hospitalisation for AF/AT recurrence, worsening of heart failure, cardioembolic event, or major bleeding) as per-protocol analysis**





Fine-Grey curves are plotted for the Surgery Group (red) and Hybrid Group (black). All-cause death was used as a competing risk event to assess the difference between the Surgery Group (blue) and the Hybrid Group (green).

HR, hazard ratio; CI, confidence interval

**Figure S5**. **Decomposition of the primary clinical endpoint as per-protocol analysis**


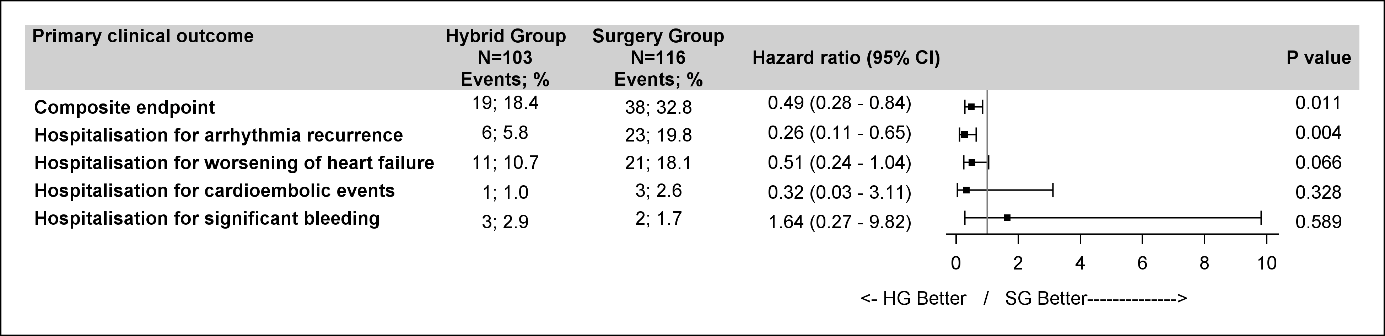


The forest plot derived from Cox regression analysis shows hazard ratio estimates (squares) with 95% CI (horizontal bars) for the treatment effects (Hybrid Group vs. Surgery Group) for the composite primary clinical endpoint and individual components of the primary clinical endpoint. Data are not adjusted for multiple comparisons. Absolute and relative frequencies of the respective events are also shown.

CI, confidence interval; HG, hybrid group; SG, surgery group

**Figure S6. Subgroup analysis for the primary clinical endpoint as per-protocol analysis**


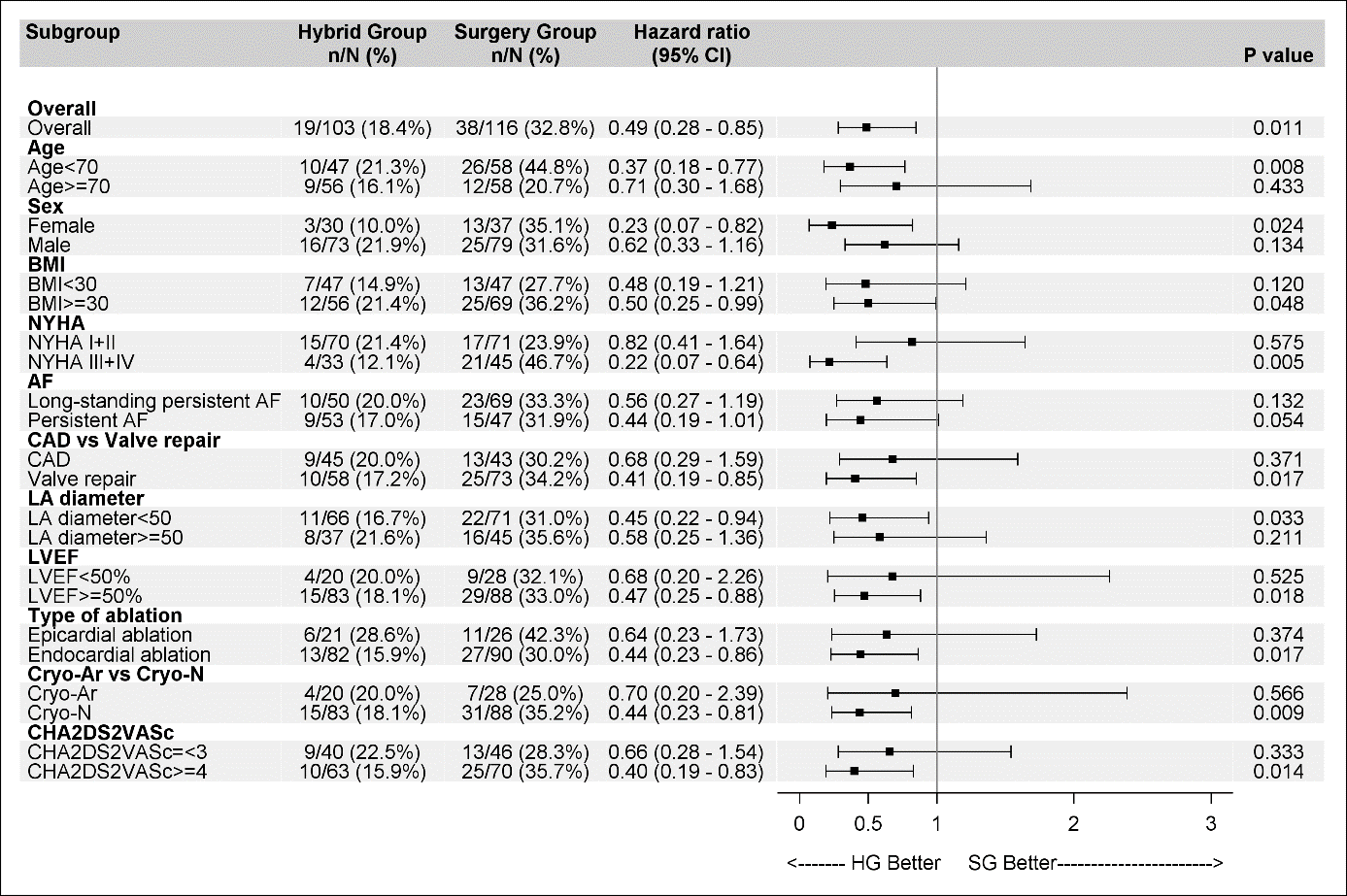


N, number of all patients; n, number of patients with the event

The forest plot derived from Cox regression analysis shows hazard ratio estimates (squares) with 95% CI (horizontal bars) for the treatment effects (Hybrid Group vs. Surgery Group) in prespecified subgroups. The widths of the CI and P-values are not adjusted for multiple comparisons.

AF, atrial fibrillation; BMI, body mass index; CAD, coronary artery disease; CI, confidence interval; Cryo-Ar, argon-based cryoablation; Cryo-N, nitrogen-based cryoablation; HG, hybrid group; LA, left atrium; LVEF, left ventricular ejection fraction; NYHA, New York Heart Association classification of heart failure; SG, surgery group.

**Figure S7.** **Hazard ratios for secondary clinical endpoints as per-protocol analysis**


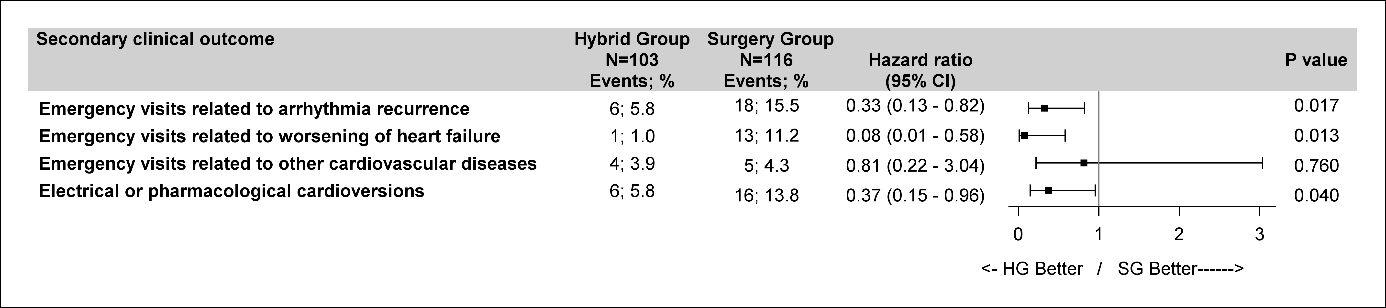


The forest plot derived from Cox regression analysis shows hazard ratio estimates (squares) with 95% CI (horizontal bars) for the treatment effect (Hybrid Group vs. Surgery Group) for individual secondary clinical endpoints. Data are not adjusted for multiple comparisons. Absolute and relative frequencies of the respective events are also shown.

CI, confidence interval; HG, hybrid group; SG, surgery group

**Study definition of clinical events requiring hospitalisation**

Arrhythmia recurrence

Atrial fibrillation, atrial tachycardia, or typical atrial flutter identified on the 12-lead surface ECG accompanied by severe symptoms precluding ambulatory treatment.

Worsening of heart failure

Signs and symptoms of congestive heart failure that is not treatable by ambulatory IV diuretics and requires any of the following: (1) continuous IV diuretic therapy, (2) IV inotropic support, (3) continuous IV nitrate infusion, or (4) in-hospital treatment of conditions that contribute to the heart failure.

Major bleeding

Major bleeding is symptomatic, requires hospitalisation, and is associated with one or more of the following conditions: (1) decrease in haemoglobin of ≥ 20 g/l over 24 hours, (2) transfusion of ≥ 2 units of packed red blood cells, (3) occurring in a critical part of the body (symptomatic intracranial, intraspinal, intraocular, pericardial, intraarticular, intramuscular with compartment syndrome, retroperitoneal).

A relevant symptomatic intracranial haemorrhage, which includes subdural, epidural, subarachnoid, and intracerebral haemorrhage, is defined as a haemorrhage that leads to a clinical worsening and hospitalisation and is assessed by the treating physician to be likely the cause of the new neurological symptom or the death. Intracerebral haemorrhage due to trauma will not be considered. For all other organs: for bleeding (e.g., gastrointestinal) in a critical area or organ to be classified as significant, it must be associated with a symptomatic clinical presentation.

Cardioembolic event

Two cardioembolic events were recognised: (A) ischemic stroke or transitory ischemic attack and (B) systemic embolism.

An ischemic stroke is defined as a new sudden focal neurological deficit of presumed cerebrovascular aetiology that persisted beyond 24 hours and was not due to another identifiable cause. A transient ischemic attack is defined as a transient episode of neurologic dysfunction caused by focal brain, spinal cord, or retinal ischemia without cerebral infarction on imaging. It is not judged as a stroke). Brain imaging should exclude haemorrhagic aetiology.

Systemic embolism is defined as abrupt vascular insufficiency associated with clinical or radiological evidence of arterial occlusion of the extremity or organ other than the brain in the absence of other likely mechanisms (e.g., atherosclerosis, instrumentation, or trauma).

Cardiovascular death

Cardiovascular death is defined as any death that is due to any of the following cardiac or vascular causes: (1) sudden cardiac death, (2) cardiac mechanical/pump failure, (3) ischemic stroke, (4) haemorrhagic stroke, (5) major bleedings, (6) systemic embolism, (7) myocardial infarction, and (8) other vascular causes.

**Study definition of clinical events not requiring hospitalisation**

Arrhythmia recurrence

Atrial fibrillation, atrial tachycardia, or typical atrial flutter identified on the 12-lead surface ECG accompanied by maximally moderate symptoms, which are manageable by ambulatory treatment, usually requiring any of the following: (1) initiation or adjustment of rate control medication, (2) initiation or adjustment of rhythm control medication, and (3) pharmacological or electrical cardioversion.

Worsening of heart failure

Signs and symptoms of congestive heart failure that are manageable by ambulatory treatment, usually requiring any of the following: (1) initiation or dose escalation of the loop diuretics, (2) treatment of arterial hypertension, (3) initiation or adjustment of the optimum medical therapy for the treatment of heart failure, i.e., angiotensin-converting enzyme inhibitors or angiotensin receptor blockers, beta-blockers, mineralocorticoid receptor antagonists, angiotensin receptor-neprilysin inhibitor, or sodium-glucose cotransporter-2 inhibitors.

Other cardiovascular diseases

Any of the following signs or symptoms: (1) hypotension, (2) syncope or presyncope spells, (3) palpitations, (4) peripheral oedema, (5) chest pain (extracardiac origin must be excluded), or (6) decreased exercise tolerance due to dyspnoea (extracardiac origin must be excluded).
