# Supplementary figures and images for: Sequential hybrid ablation vs. surgical CryoMaze alone for treatment of atrial fibrillation: results of multicentre randomized controlled trial
Source: Europace. 2024 Feb 2;26(2):euae040. doi: 10.1093/europace/euae040 (PMC10872694; doi:10.1093/europace/euae040)

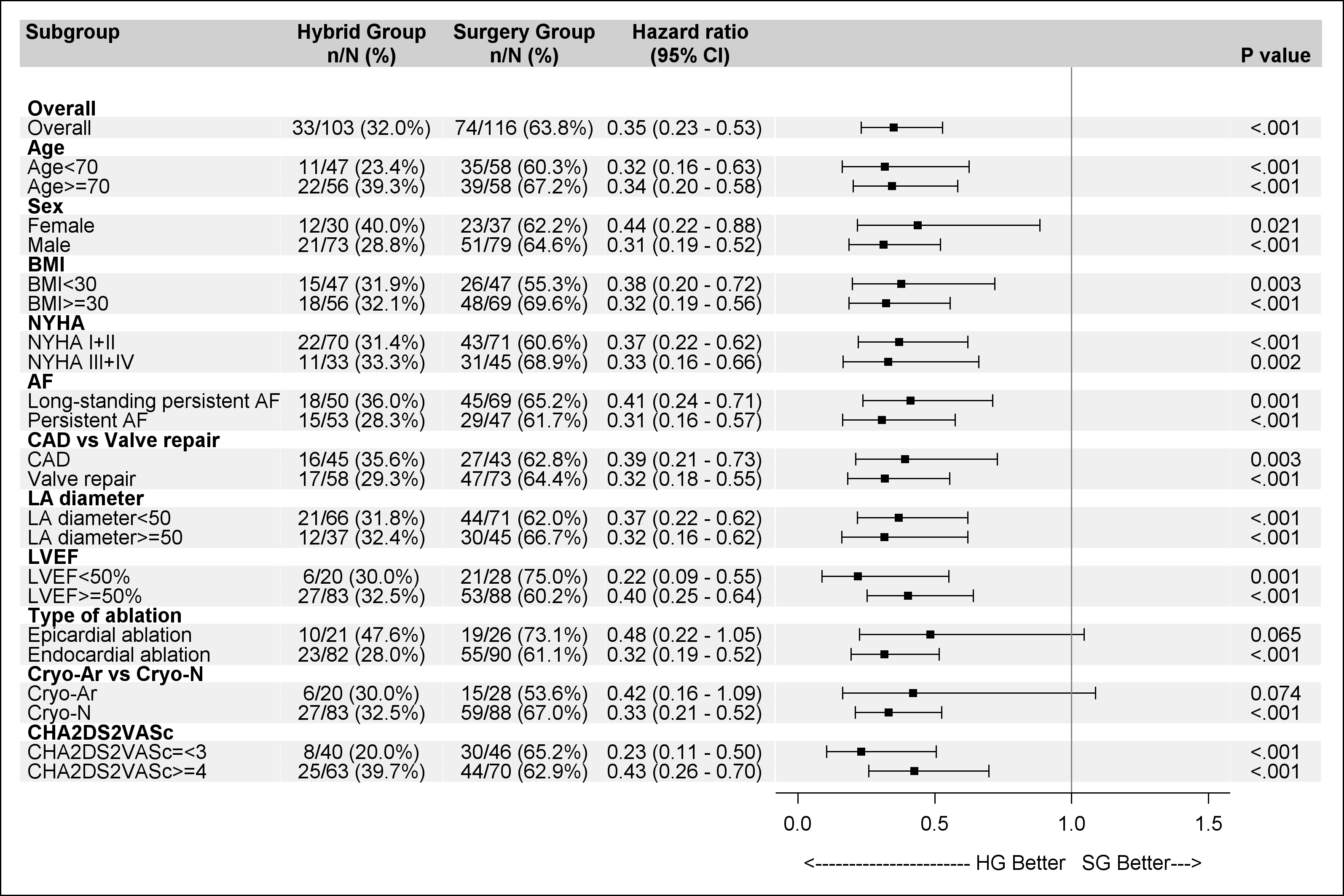

Supplement: euae040_Supplementary_Data [file euae040_supplementary_data.zip › Figure S3 Surhyb EHJ PP FP efficacy_revised.png]

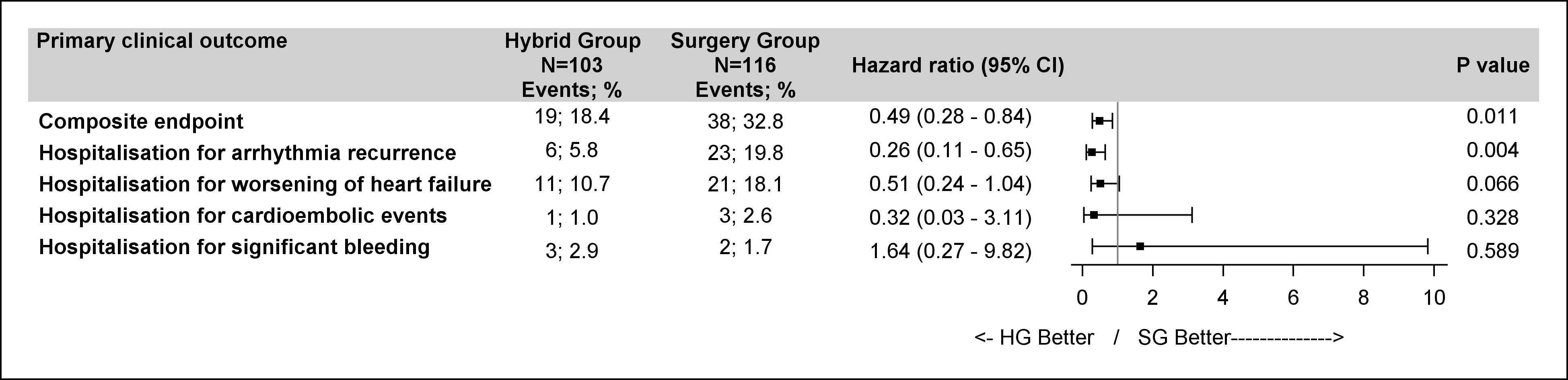

Supplement: euae040_Supplementary_Data [file euae040_supplementary_data.zip › Figure S5 Surhyb EHJ PP FP clinical decomposition.tif]

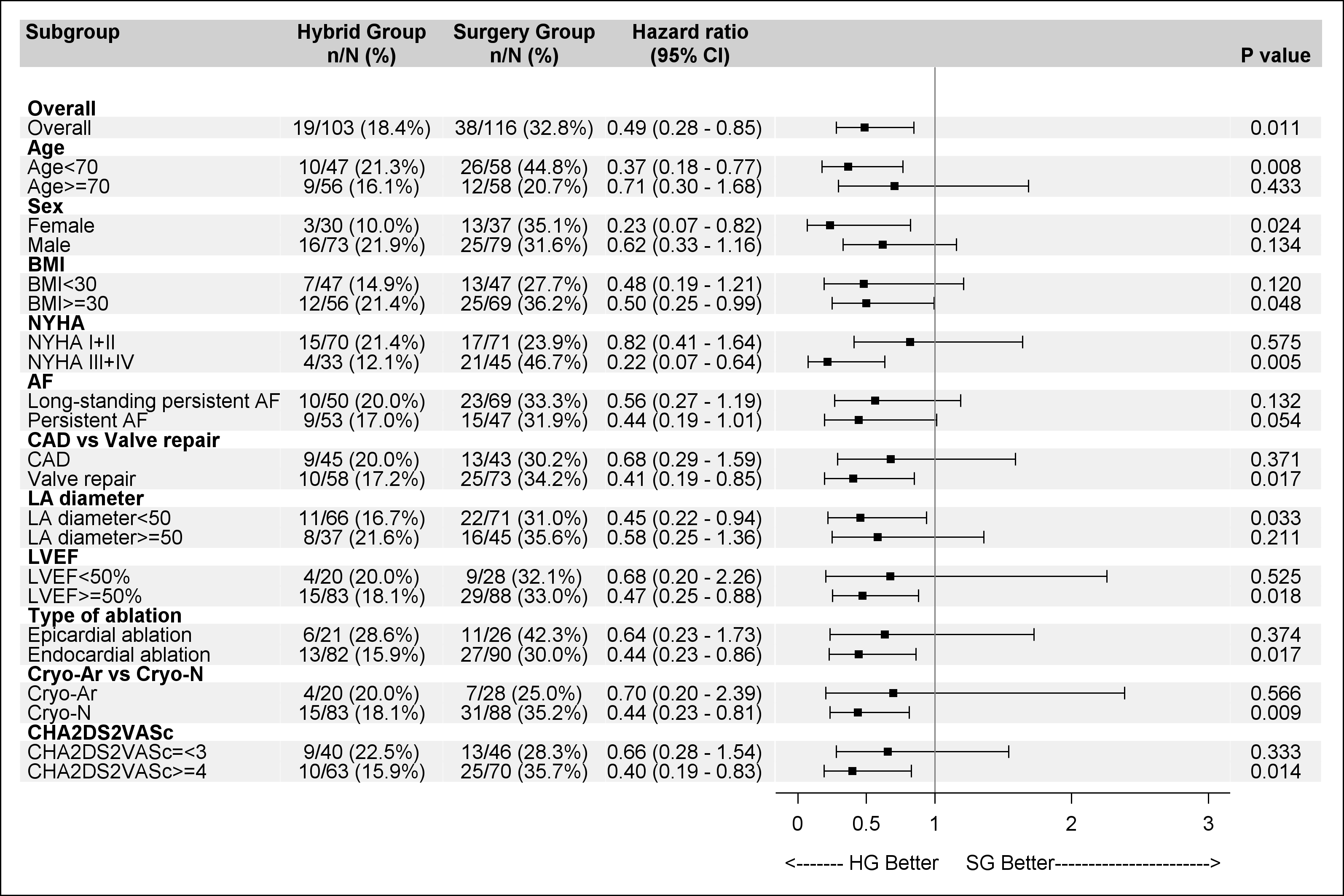

Supplement: euae040_Supplementary_Data [file euae040_supplementary_data.zip › Figure S6 Surhyb EHJ PP FP clinical subgroups_revised.png]

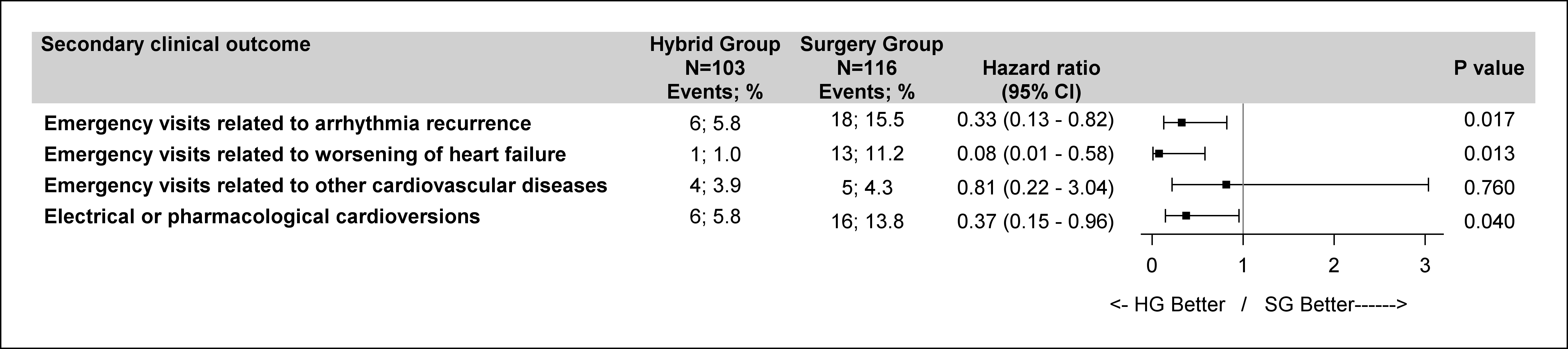

Supplement: euae040_Supplementary_Data [file euae040_supplementary_data.zip › Figure S7 Surhyb EHJ PP FP secondary clinical.tif]

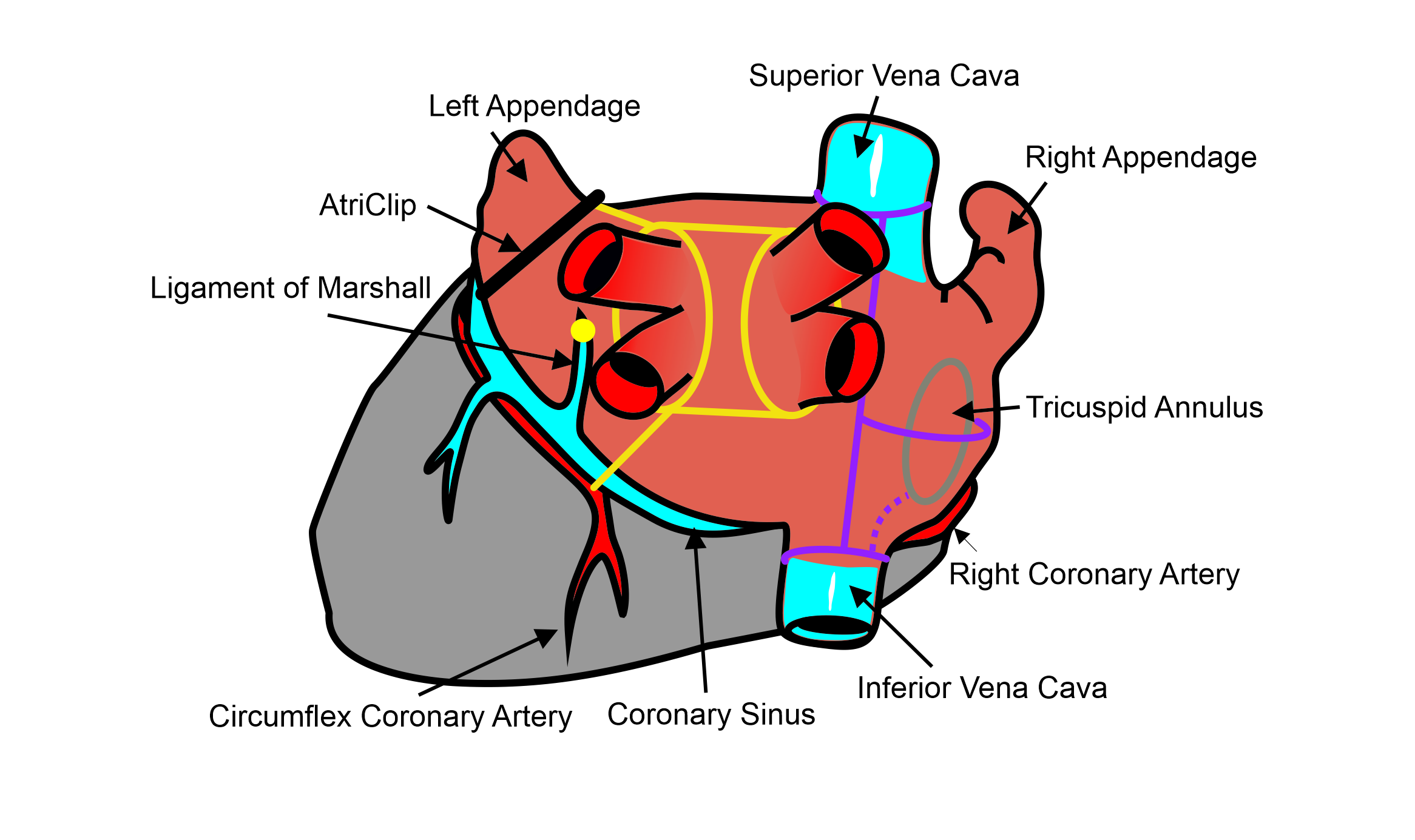

Supplement: euae040_Supplementary_Data [file euae040_supplementary_data.zip › Figure S1 Surhyb EHJ lesions.tif]
